# Real-Time analysis of exosome secretion of single cells with single molecule imaging

**DOI:** 10.32604/biocell.2021.017607

**Published:** 2021-09-01

**Authors:** Pengfei ZHANG, Shaopeng WANG

**Affiliations:** 1 Biodesign Center for Bioelectronics and Biosensors, Arizona State University, Tempe, 85287, USA; 2 School of Biological and Health Systems Engineering, Arizona State University, Tempe, 85287, USA

**Keywords:** Plasmonic scattering, Label-free, Single molecule imaging, Cell secretion

## Abstract

The exosome-mediated response can promote or restrain the diseases by regulating the intracellular pathways, making the exosome become an effective marker for diagnosis and therapeutic control at the single-cell level. However, real-time analysis is hard to be achieved with traditional approaches because the exosomes usually need to be enriched by ultracentrifugation for a measurable signal-to-noise ratio. Recently developed label-free single-molecule imaging approaches may become an real-time quantitative tool for the analysis of single exosomes and related secretion behaviors of single living cells owing to their extreme sensitivity.

## Introduction

Exosomes are released by various mammalian cells, and they can be captured by distant cells, where they can alter the biological response of recipient cells. Intercellular communications via exosomes are involved in many physiological derangements, such as neurodegeneration, tumor mutations, and inflammatory reactions. The exosomes-mediated response can promote or restrain the diseases by regulating the intracellular pathways, making the exosome become an effective marker for diagnosis and therapeutic control (Kalluri and LeBleu, 2020). In addition, the exosomes can be engineered to direct various therapeutic cargos to target cells to enhance the bioavailability and minimize side effects. Recent study also shows that the prokaryotic cells, such as bacteria, also release membrane vesicles owning similar size and functions to exosomes, and these exosome-like vesicles can affect the cell behaviors, such as drug resistance, during acquired abnormalities (Toyofuku *et al*., 2019). Despite the great potential of exosomes and exosome-like vesicles in biological research and disease treatments, characterizing the exosomes and related cell secretion processes is still a challenging task. The exosomes usually have the size down to ~30 nm, and some exosome-like vesicles have the diameter of only ~20 nm, which is close to the size of single protein molecules, making it challenging to study them with conventional optical detection technologies. The traditional highly sensitive imaging methods, such as surface plasmon resonance and epi fluorescence microscopies, can only effectively analyze the exosomes with size over 100 nm (Bos *et al*., 2021; Yang *et al*., 2020). However, it is pointed out that the solid meshwork of the cells can restrict the diffusion of particles with size over 75 nm (Etoc *et al*., 2018), and the exosomes with size smaller than 75 nm may play more important role in the long-distance cell communication and crowded cell environment. Besides, real-time analysis is hard to be achieved with these traditional approaches because the exosomes usually need to be enriched by ultracentrifugation for measurable signal to noise ratio.

### Main text

Single molecule imaging was firstly developed in the late 1980s by employing the fluorescence emission to shift detection wavelength away from excitation wavelength, and dichroic mirror and optical filter were used to achieve near-zero background for detection of the weak signals from single molecules. Single molecule fluorescence microscopy allows detection and tracking of single proteins, and its feasibility for small exosome detection has been demonstrated owing to its extreme sensitivity (Lee *et al*., 2018). This indicates that single molecule imaging is capable for single exosome detection. Recently, label free single molecule imaging approaches, such as interferometric scattering (Young *et al*., 2018), photonic crystal resonant scattering (Li *et al*., 2021) and plasmonic scattering imaging (Zhang *et al*., 2020; Zhang *et al*., 2021), are developed for single molecule detection by measuring the natural Rayleigh scattering instead of the fluorescence emission. The label free single molecule imaging provides the quantitative measurement of the intrinsic molecular properties, such as molecular size and mass, allows the long-term precise monitoring of molecular interaction for kinetic analysis without the influence of photobleaching, photoblinking or limited excited-state lifetimes, and avoids the tracking error caused by nonuniform distribution of fluorescent labels. These abilities will enable analysis of single exosome heterogeneity, quantitative measurement of exosome intrinsic properties and study of exosome-cell interaction processes at a level of detail. Furthermore, the label free optical imaging approaches allow measuring the objects in their native forms, thus providing an easy solution for real-time analysis of cell secretion.

**FIGURE 1.**
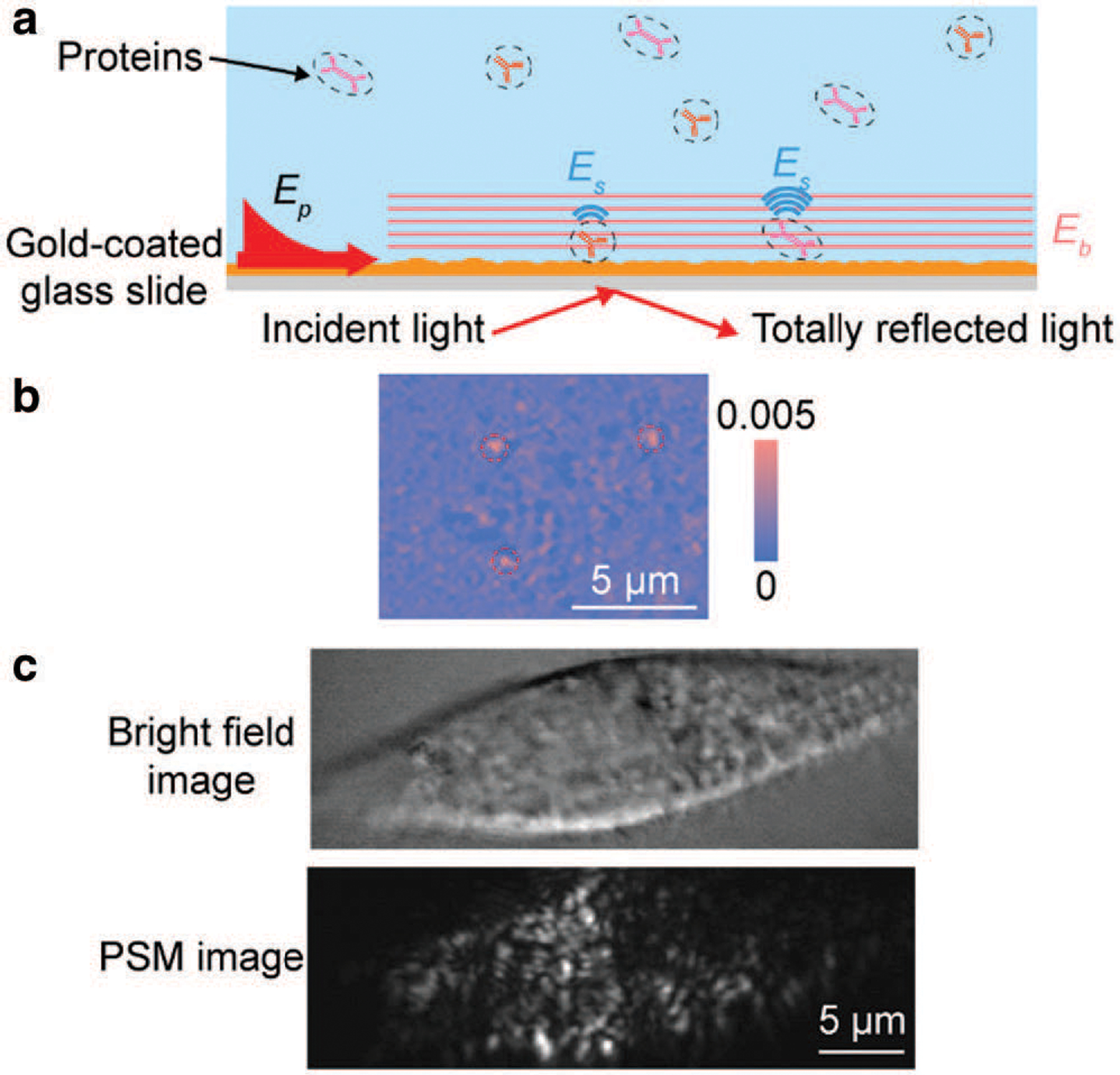
(a), Simplified sketch of the PSM imaging system, where surface plasmonic waves (Ep) are excited by light from the bottom of a gold-coated glass slide and scattering of the plasmonic waves by a particle or protein (E_s_) and by the gold surface (E_b_) is collected from the top to form a PSM image. (b), Three IgG proteins (molecular weight = 150 kDa) imaged by PSM. Red dashed circles indicated the binding positions of the proteins. (c), One RBL-2H3 cell imaged by bright field and PSM microscopies, where the focal adhesions, the physical mediators linking the cell cytoskeleton with extracellular matrix, can be clearly observed as bright spots in PSM image.

Plasmonic scattering microscopy (PSM), one kind of label free single molecule imaging approach, was recently developed based on the conventional surface plasmon resonance (SPR) system (Fig. 1a) (Zhang *et al*., 2020; Zhang *et al*., 2021). PSM employs the scattering of surface plasmonic wave by gold film surface roughness as reference field for interference imaging of single molecules. The surface plasmonic wave propagates within ~100 nm from the SPR sensor surface, making it immune to interference of molecules and impurities in the bulk solution for low detection noise. In addition, with large plasmonic enhancement (20–30 times), PSM can achieve the same signal-to-noise ratio with wider field of view compared with non-plasmonic methods, such as interference scattering microscopy (Young *et al*., 2018). Owing to the great signal to noise ratio, PSM can image the single proteins with molecular weight down to 150 kDa and size down to ~10 nm (Fig. 1b). This indicates that it can be employed to image single exosome or exosome-like vesicle with molecular weight down to several thousand kDa and size down to ~20 nm. In addition, PSM image has no parabolic tail as the conventional evanescent imaging technologies, such as SPR and total internal reflection microscopies, thus allowing imaging of cell membrane with high spatial resolution (Fig. 1c). This indicates that it allows high precision tracking of interactions between single exosomes and cell membrane. Furthermore, the evanescent illumination, limiting the strong illumination light (~kW/cm^2^ or higher) to be within the ~100 nm of the sensor surface, thus allowing the implementation of simultaneous fluorescence imaging for detection most of the cell volume without worry of quick photobleaching. This means that the PSM permits the flexible combination with other optical imaging approaches, thus allowing multiplexed detection of exosome secretion process of single cells.

There are still a few challenges need to be overcome for stable real-time analysis of exosome secretion with PSM. The first challenge is that the cells may release many products during the secretion and label free imaging is sensitive to all objects with the size down to single protein level, so the recognition of target exosomes need to be addressed. A potential solution for this challenge is modifying the sensor surface with antibodies targeting exosome surface markers, such as CD63 and CD81, for recognizing the exosomes specifically (Kalluri and LeBleu, 2020). The second challenge is that the cells create much larger scattering signal intensity than exosomes, so different experimental parameters, including incident intensity and camera exposure time, are required for the imaging of cells and exosomes, respectively. This puts forward high requirements on the optical arrangements of the system. The third challenge is that the single cells usually release the exosomes after corresponding stimulation. Adding the stimulation to local target cells precisely is required for identify exosome secretion from single cells. Further optimization on the optical system and related protocols is still required for introducing PSM and other label free single molecule imaging approaches into the study of exosome secretion of living cells.
